# *Porphyromonas gingivalis*, Periodontal and Systemic Implications: A Systematic Review

**DOI:** 10.3390/dj7040114

**Published:** 2019-12-11

**Authors:** Luca Fiorillo, Gabriele Cervino, Luigi Laino, Cesare D’Amico, Rodolfo Mauceri, Tolga Fikret Tozum, Michele Gaeta, Marco Cicciù

**Affiliations:** 1 Department of Biomedical and Dental Sciences, Morphological and Functional Images, University of Messina, Policlinico G. Martino, Via Consolare Valeria, 98100 Me, Italy; gcervino@unime.it (G.C.); cdamico@unime.it (C.D.); mgaeta@unime.it (M.G.); mcicciu@unime.it (M.C.); 2 Multidisciplinary Department of Medical-Surgical and Dental Specialties, Second University of Naples, 80100 Naples, Italy; luigi.laino@unicampania.it; 3 Department of Surgical, Oncological and Oral Sciences, University of Palermo, 90127 Palermo, Italy; rodolfo.mauceri@unipa.it; 4 Department of Periodontics, College of Dentistry, University of Illinois at Chicago, Chicago, IL 60613, USA; ttozum@uic.edu

**Keywords:** *Porphyromonas gingivalis*, periodontitis, bacteremia, LPS, brain diseases, neurodegenerative diseases, cardiovascular diseases, arthritis, oral health

## Abstract

In recent scientific literature, oral infections and systemic manifestations, or correlations between oral health and systemic diseases are a topic of discussion. *Porphyromonas gingivalis* is one of the bacteria implicated in the biofilm formation of bacterial plaque, and plays an important role in the progression of periodontal disease. In this systematic review authors have evaluated the literature of the last 10 years on *P. gingivalis* and all the systemic implications proven. This study therefore evaluates all the districts of the organism in which this bacterium may have implications. From the results it emerges that *P. gingivalis* has implications in the onset of different systemic pathologies, including rheumatoid arthritis, cardiovascular pathologies, and neurodegenerative pathologies. Surely, understanding the mechanisms of diffusion of this bacterium, it would be possible to prevent a series of pathologies. Thus, putting the dentist clinician at the center of prevention for these diseases.

## 1. Introduction

### 1.1. Rationale

Oral health has an important role with regard to systemic health, this is reflected in the scientific literature. There are numerous correlations between problems or oral alterations and systemic health of the patient [1,2,3,4,5]. In particular, the implications of chronic oral inflammation, such as periodontitis, may be different [6]. Having both an influence on the course of oral and general conditions. The correlations do not seem to be very clear, and the literature on the subject is numerous. Periodontal disease includes gingivitis and periodontitis, pathologies that recognize an essentially bacterial multifactorial aetiology with the interaction of three cofactors:

- Host susceptibility;
- Environmental factors;
- Behavioral factors.

Bacterial plaque, although a necessary condition, is affected by the indispensable role of interaction with the host. Numerous local and systemic factors (e.g., diabetes) influence the clinical course. Gingivitis affects the gum close to the tooth (marginal gingiva) and is characterized by reddening of the gingival margin, edema, bleeding under mechanical stimulation, and sometimes by volume increases. These are completely reversible clinical pictures if adequately treated. Periodontitis is a group of pathologies that have in common the destruction of the tooth support system. They manifest themselves with a loss of attachment and bone, pocket formation, and gum recession. They are always preceded by gingivitis and, therefore, if the latter is prevented, it is possible to prevent far more serious periodontitis.

The characteristic sign of periodontitis is the formation of the periodontal pocket associated with tooth mobility. In recent years, much research has focused on the mechanisms that underlie periodontal disease, and in particular on the condition of balance that is created between the immune system and bacterial biofilm. One of the bacteria present in the oral plaque biofilm is *Porphyromonas gingivalis* (*P. gingivalis*) [7]. *P. gingivalis* belongs to the phylum Bacteroidetes and is a nonmotile, Gram-negative, rod-shaped, anaerobic, pathogenic bacterium. It forms black colonies on blood agar when prelevated in the respiratory tract and the colon, it is implicated in periodontal disease [8,9]. It has been isolated from women with bacterial vaginosis and on brain biopsies of patients with Alzheimer’s disease too [10,11,12,13].

### 1.2. Objectives

The aim of this study is to evaluate a correlation between *P. gingivalis* periodontal implications and any proved correlated systemic disease.

## 2. Materials and Methods

### 2.1. Protocol and Registration

This manuscript has been registered as a Review in a Systemic Review database called PROSPERO. It is an International Prospective Register of Systematic Reviews about health and social care. Obtained PROSPERO registration number is 145886 on 04/08/2019. The main question of this study was elaborated following the PICOT (Population/Intervention/Comparison/Outcome/Time) study design [14].

This revision paper followed a protocol according to PROSPERO and according to PRISMA (Transparent Reporting of Systematic Reviews and Meta-Analyses) [15,16,17].

### 2.2. Eligibility Criteria

The results obtained from the literature search were filtered, through software and manual screening, according to these inclusion and exclusion criteria:

- Inclusion Criteria:
  ○ Human studies;
  ○ Information about *P. gingivalis* and periodontal implication;
  ○ Information about *P. gingivalis* and systemic disease;
  ○ In vitro and in vivo studies about *P. gingivalis;*
  ○ Last 10 years studies.
- Exclusion Criteria:
  ○ In silica studies;
  ○ Not on human studies;
  ○ Not in English;
  ○ Not accessible title or abstract;
  ○ Not enough information about main question of review.

### 2.3. Information Sources

Results have been obtained after a research and a screening on scientific international database as PubMed, Embase, and Web of Science. Automatic filter and platforms software have been used for results screening.

### 2.4. Search

Search has been conducted using this keyword on information sources: “*Porphyromonas gingivalis*” and “Systemic”. Search date is 01/07/2019.

The database search protocol details is: “*Porphyromonas gingivalis*” (All Fields) and “systemic” (All Fields) and (Review (ptyp) and “loattrfull text” (sb) and “2009/07/01” (PDat): “2019/07/01” (PDat) and “humans” (MeSH Terms) and English (lang)).

**Figure 1:**
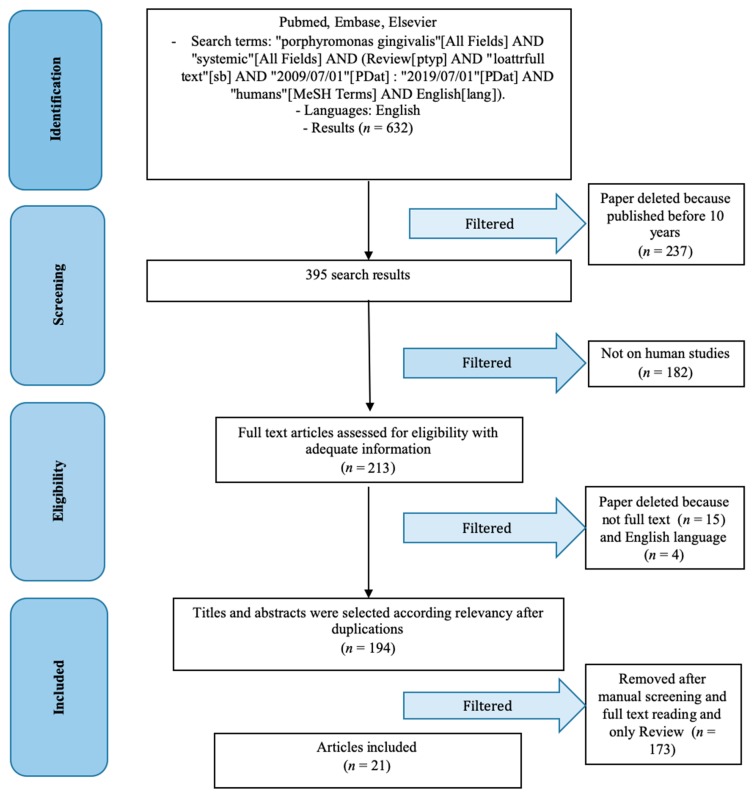
PRISMA flow chart.

Keyword were chosen by authors after a discussion with the aim to include a larger as possible number of results (Figure 1).

Population, Intervention, Comparison, Outcome (PICO) questions were:

- Are patients who have periodontitis an increased for systemic health status compared with patients without periodontitis?
- Does *P. gingivalis* influence systemic health in patients who have periodontitis?

### 2.5. Study Selection

The selection process was conducted by authors with the aim to include relevant studies for this review. After the electronic eligibility criteria applying, authors conducted a manual study selection independently. Selection of study conducted to this manuscript.

### 2.6. Data Collection Process

Data collection process has been conducted independently by two authors of two different University (L.F. University of Naples and G.C. University of Messina). After data screening completion they clarified any doubt with another two expert reviewers (M.C. and L.L.). The original draft then was revised by a last independent author (T.T.).

### 2.7. Data Items

The following data items were considered during data collection:

- Summary of items (Table 1):
  ○ Neurology;
  ○ Cardiology;
  ○ Immunology;
  ○ Rheumatology;
  ○ Diabetology;
  ○ Oncology;
  ○ Biology.
- Investigated data items on articles (Tables 2 and 3):
  ○ Authors and year–article authors and year of publication (reference have been added);
  ○ Item–article and authors evaluated items;
  ○ Outcome–main results of the study;
  ○ Medical disease–investigated medical disease.

### 2.8. Risk of Bias in Individual Studies

The grade of bias risk was independently considered, and in duplicate by the two independent reviewers at the moment of data extraction process. This revision followed the Cochrane Collaboration’s two-part tool for assessing risk of bias and PRISMA statement [15,16].

Potential causes of bias were investigated:

- Selection bias;
- Performance bias and detection bias;
- Attrition bias;
- Reporting bias;
- Examiner blinding, examiner calibration, standardized follow-up description, standardized residual graft measurement, and standardized radiographic assessment.

In this way, the possible random sequence generation, the possible allocation concealment, the possibility of blinding of participants and personnel, the possible presence of having incomplete outcome data and other biases were all considered and evaluated.

This method applied by the two reviewers was valuable for giving to each study a level of bias. Then, the selected papers were classified with low, moderate, high and unclear risk.

### 2.9. Summary Measures

Accordingly to PRISMA statement, principal summary of measures has been showed in Table 1.

### 2.10. Synthesis of Results

The summary of the results was carried out manually by the authors, after reading the title, abstract and full text of each article.

### 2.11. Risk of Bias across the Studies

Risk of bias across the studies has been evaluated according to PRISMA guidelines. Limitation of detailed design, inconsistency, indirectness, imprecision, and publication bias was evaluated during this workflow.

### 2.12. Additional Analysis

Additional information about periodontitis and *P. gingivalis* was performed. A literature search provided results about *P. gingivalis* biology and periodontitis physiopathology.

## 3. Results

### 3.1. Study Selection

Results were obtained according to the Materials and Methods instructions. Obtained results were 632, using paragraph 2.4 keywords. Subsequently, according to eligibility criteria, results were screened. Authors decided to maintain only the last 10 years results, because of novelty and accuracy on the diagnostics method, and quality of results due to new technologies, 395 results remained after this first screening. Only 213 studies on human have been considered, and authors analyzed full text (198) and English articles (194). The last step to complete study results selection was to compare only the review articles type. Only 21 results, after a reading and a manual authors screening resulted eligible for this study (Figure 1).

### 3.2. Study Characteristics and Results of Individual Studies

Results of individual studies are listed below in Table 2.

### 3.3. Synthesis of Results

**Figure 2:**
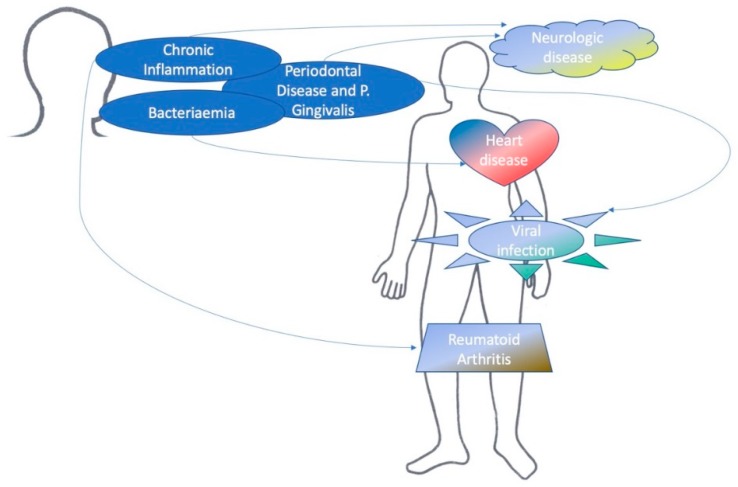
Periodontal disease and systemic diseases (for kind concession of Dr. Luca Fiorillo).

According to Table 1 and Table 3, authors defined a scheme to better clarify periodontitis systemic correlations (Figure 2) [39].

### 3.4. Risk of Bias

A risk of bias analysis has been performed according to PRISMA guidelines and results have been shown in the Table 4.

### 3.5. Additional Analysis

*Porphyromonas gingivalis* is considered the second most studied parodontal pathogen, after *Aggregatibacter actinomycetemcomitans*. This is a gram-negative bacterium; it is a bacterium that is often found in the form of a coccobacterium, or rod. It is part of the group of black-pigmented Bacteroides. The bacteria of this group form black-brown colonies on blood agar plates. They were almost immediately associated with periodontal disease, within this family of asaccharolytic species (*P. gingivalis*), intermediate species and highly saccharolytic species (*Prevotella melaninogenica*). They were immediately of scientific interest as they produce a series of particularly high virulence factors. In fact, *P. gingivalis* is able to produce collagenase, a series of proteases, hemolysins, endotoxins, fatty acids, ammonia, hydrogen sulfide, indole and others; furthermore, this bacterium is able to inhibit the polymorphonuclear migration through the endothelial barrier. This species is present in patients with periodontal disease, but the quantities are reduced, compared to patients who have aggressive forms of this disease. *P. gingivalis* is able to attack epithelial cells of the gingival mucosa, and endothelial cells. This fact can be found above and within the epithelial cells, due to the use of fimbriae by the bacterium. Following tooth brushing and the formation of the glycoprotein of dental enamel, a bacterial biofilm starts to form on it. Among the first colonizers we certainly recognize the *Streptococcus oralis* and *Streptococcus mitis*; *Streptococcus gordonii* and *Streptococcus oralis*; and *Streptococcus* *sanguis*. Subsequently a complex process of bacterial aggregation takes place up to the *Fusobacterium nucleatum,* which guarantees, directly or through *Treponema denticola*, the adhesion of *P. gingivalis*. *P. gingivalis* is a late colonizer, like *Actinomyces actinomycetemcomitans* or the intermediate *Prevotella* [40]. It should also be emphasized that the causes of periodontal disease can be multiple. Some factors, such as biomechanical factors, may have an important role in the course of this multi-factorial pathology [41,42,43,44].

## 4. Discussion

### 4.1. Summary of Evidence

During the analysis of the results we carefully extrapolated all the results, as evident in the previous section, and we also evaluated the conclusions, and future perspectives of each article. These are reported below, and were discussed by the authors, according to Table 1 scheme.

#### 4.1.1. Neurology

According to Dominy et al. [10] *P. gingivalis* has been identified on Alzheimer’s patients histologies. Toxic proteases, called gingipains have been found too. Gingipains are neurotoxic, and its inhibition reduces the bacterial load of *P. gingivalis*. The small-molecule inhibitors designed by authors, could inhibit gingipains and so block Aβ1–42 (component of amyloid plaques) production by *P. gingivalis*. Inhibiting *P. gingivalis* can reduce neurotoxicity, and this inhibitor could be valuable for treating brain colonization. According to We et al. [24] PD and chronic systemic inflammation could be correlated with neuroinflammation and AD. Periodontitis can be correlated to AD and other neurodegenerative diseases.

#### 4.1.2. Cardiology

According to Bale et al. [18] periodontal disease can influence the atherosclerosis pathogenesis triad; PD is caused by high-risk pathogens for arterial disease, so dental community should manage PD reducing so cardiovascular disease that is one cause of morbidity and mortality on general population. According to Chistiakov et al. [21] there is an important correlation between PD and CVD. All periodontal disease characteristics as bacterial toxins, virulence factor, and immunological alteration could develop CVD. In summary, clinicians could prevent CVD and heart disease just preventing PD and PD products. Periodontal therapy could be the first step on cardiovascular disease therapy [45]. According to authors, a dentist should be on the first line for CVD therapy and prevention. In another review study of Alfakry et al. [22] a coronary heart disease correlation has been showed. According to authors, PD and endogenous degradation mechanisms could lead to tissue destruction. According to Reyes et al. [29] despite no bacteria have been found on atheromas, periodontal bacteria could diffuse through systemic vascular tissue, and cause atherosclerosis. Periodontal bacteria isolated from human atheromas did not cause disease on animal models, not respecting Koch postulates. Preventing PD and periodontal chronic inflammation could be a first therapeutic step for atherosclerosis interdiction. According to Huck et al. [34] periodontal disease and *P. gingivalis* infection could lead to atherosclerosis progression. There is a link between these pathologies, and periodontal treatment could be a benefit to systemic condition.

According to McNicol and Israels [35] bacteriaemia is frequent in PD patients, and it is the basis of atherothrombosis development. In their study they evaluate the bacteria ability to activate platelets. According to Inaba and Amano [36] oral bacteria play an important role on systemic disease. Hayashi et al. [37] evaluated different systemic diseases correlated to periodontal disease and *P. gingivalis* infection. *P. gingivalis* can induce chronic inflammation and maintain this status at sites distant from oral infection.

In a recent publication from the study of coronary diseases and periodontal disease, it was shown that high levels of the group of periodontal pathogens defined as the sum of five pathogens examined (*Actinomyces actinomicetemcomitans*, *Tannerella forsythia*, *P. gingivalis, Prevotella intermedia,* and *Treponema denticola*) and high levels of colonization of *A. actinomicetemcomitans* were significantly associated with cardiovascular disease in a correct multivariate analysis. It has also been shown that endothelial cells can be damaged by the ability of *P. gingivalis* to adhere, invade and proliferate in endothelial coronary cells. It is possible that this phenomenon may interfere with the physiological function of vasodilation due to the damage caused to both endothelial and smooth muscle cells. When in vitro data were translated into a cohort study, this association was further strengthened and it was shown that patients with periodontitis had significant impairment of flow-mediated dilation [2,4,10,39,40,41,42,43,44,45,46].

#### 4.1.3. Rheumatology

Scher et al. [26] showed how there is a correlation between some immunological systemic disease and PD. This should be caused by circulating bacterial antibodies that lead to systemic inflammation. PD has been correlated with RA and smoking habits. Han et al. [31] showed a large list of systemic alteration due to oral microbioma and periodontal infections. Adverse pregnancy outcomes, rheumatoid arthritis, inflammatory bowel disease and other pathologies, could be caused by *P. gingivalis*, *Streptococcus mutans*, *Campylobacter rectus,* or *Fusobacterium nucleatum*. *P. gingivalis* is one of the extra-oral prone bacteria. Evaluate and quantify extraoral pathogens levels could predict disease potential. According to Detert et al. [38] rheumatoid factor and the anticyclic citrullinated peptide antibody are present in PD and RA. *P. gingivalis* plays a role on citrullination.

#### 4.1.4. Diabetology

According to El-Shinnawi and Soory [32] PD affected patients present other inflammatory condition as CHD, RA, or insulin resistance. For example, IL-1, IL-6, and TNF-alpha that are linked to periodontal disease progression, are associated to insulin resistance too. Periodontal treatment could be essential for systemic inflammation prevention.

#### 4.1.5. Oncology

Gholizadeh et al. [20] in their review evaluated the correlation between oral chronic inflammation and OC development [47,48,49]. *P. gingivalis* and other microbes’ species are implicated on PD, which could be an etiological factor in certain cancers. A multicenter study of 405 cases of pancreatic cancer and 416 controls found that the titers of anti-*P. gingivalis* antibodies were higher in patients with pancreatic cancer than in healthy subjects, suggesting that the oral microbiota, in particular that of the periodontal patients, may be considered a potential biomarker of human diseases. A statistically significant association between squamous cell carcinoma of the head and neck and periodontal disease has also been reported, suggesting that periodontal disease may be an independent risk factor, with practical implications for prevention, early diagnosis, and treatment, in the search for a possible improvement in the prognosis of the disease. Understanding host-microbe interaction and related cause-effect mechanisms has paved the way for a broader understanding of the role of the microbiome in health and disease conditions, providing new therapeutic approaches in clinical practice [47,48,49,50,51,52,53]. 

#### 4.1.6. Biology and Immunology

In a Tiantian et al. [19] study authors showed how viruses and bacteria are associated in periodontal disease [50], therefore periodontal disease and viral infection could develop systemic disease too. *P. gingivalis* and a virus interaction could be studied to provide a new idea for patient’s treatment. Xie [23] showed some biological mechanisms of *P. gingivalis*. *P. gingivalis* is one of the responsible of chronic periodontitis. Its products, as vesicles, could be the key to understand some communicative, diffusion mechanisms of *P. gingivalis* and its systemic implications. Grover et al. [27] identified *P. gingivalis* as one of the responsible for PD. Knowing all virulence factors should provide a potential antigenic target for a periodontal vaccine. 

According to Brusca et al. [28] mucosal sites exposed to bacteria represent the initial site of autoimmune generation, identify biomarkers and therapeutic approaches on RA are the future prospective of this study. Michaud et al. [30] believe that the complex interplay between host immune response, bacteria, and environmental factors could lead not only to gastric cancer, but to pancreatic cancer too. Bacteriaemia preventing could freeze some host immunological mechanisms. An interesting study of Imai and Ochiai [33] revealed how some periodontal bacteria could lead to HIV-1 maintenance, latency, and reactivation. Periodontal disease so could accelerate acquired immune deficiency syndrome (AIDS) in infected patients. The prevention of periodontal disease, once again, can slow down a systemic pathology and improve the outcome of these diseases.

Surely the discussion of these results makes us understand how the perspectives are important in the field of prevention and systemic pathologies. Since periodontitis has such a crucial role in the development and onset of other pathologies, the results show how much the dentist has to play a crucial role in the diagnosis, therapy, and prevention of this.

### 4.2. Limitations

The major limitation of this study was represented by the fact of not having univocal results, on which it is possible to perform a statistical analysis. The results of the review were in fact purely descriptive/epidemiological, of what the systemic pathologies connected in their etiopathogenesis to periodontal disease are or may be. Since it is a revision of reviews, it was also not possible to calculate the risk of bias on an individual basis.

## 5. Conclusions

As could be seen from the results, oral health, and in particular, oral pathology, could have important systemic repercussions. The state of constant inflammation, or even the bacterial blood circulation or bacterial products, could be a further cause of system pathologies. Preventing negative oral conditions can be a good starting point for the clinician to achieve good general health. Moreover, oral manifestations can be useful to diagnose some pathologies early, or they can represent one of the first steps of some chronic pathologies.

## Figures and Tables

**Table 1:** Table representing all periodontal and *Porphyromonas gingivalis* correlation on systemic condition.

**Neurology**	Chronic inflammation and Alzheimer’s Disease (AD)
	*P. gingivalis* and AD
**Cardiology**	Atherosclerosis risk factor and periodontal disease.
	PD and Cardiovascular Disease (CVD), Age, Smoking, Diabetes
	Chronic infection and Coronary Heart disease (CHD)
	Periodontal Bacteria and atherosclerosis
	Cytokines and coronary heart disease
**Immunology**	Periodontitis an immune response
	Antigenic target for *P. gingivalis*
	Microbiome, Periodontal disease
	Serum lipoprotein concentration, endothelial permeability, lipoprotein binding in intimate
	Periodontal disease and AIDS progression
**Rheumatology**	Rheumatoid Arthritis
	Osteoporosis
**Diabetology**	Diabetes mellitus
	Insulin resistance
**Oncology**	*P. gingivalis* and Oral Cancer (OC)
	Bacterial infection and Pancreatic Cancer (PC)
**Biology**	Virus and periodontal disease correlation
	Bacteriaemias and systemic disease
	Periodontal disease and Systemic diseases
	*P. gingivalis* biology
	Oral bacteria and extraoral infections

**Table 2:** Table of main results and item investigated on reviews.

Authors and Year	Items	Outcomes
Dominy et al., 2019 [10]	*P. gingivalis* and Alzheimer’s disease	*P. gingivalis* and gingipains were identified in the brain of Alzheimer’s patients. Gingipain inhibition reduced the bacterial load of *P. gingivalis* infection.
Bale et al., 2017 [18]	Serum lipoprotein concentration, endothelial permeability, lipoprotein binding in intima	This study supports that Periodontal disease (PD) could be associated with arterial disease
Tiantian et al., 2016 [19]	Virus and periodontal disease correlation	*P. gingivalis* can interact with a variety of bacterium viruses which may be the reason for chronic periodontitis and systemic disease
Gholizadeh et al., 2016 [20]	*P. gingivalis* and Oral Cancer (OC)	PD, systemic condition and head and neck cancer (HNC) could be correlated
Chistiakov et al., 2016 [21]	PD and Cardiovascular Disease (CVD), Age, Smoking, Diabetes	Periodontal pathogens, virulence factors and bacterial endotoxins are correlated with CVD development as endothelial dysfunction, systemic inflammation, oxidative stress, lipid accumulation, vascular remodeling, and atherothrombosis.
Alfakry et al., 2016 [22]	Chronic infection and Coronary Heart disease (CHD)	Inflammation activates endogenous degradation pathways mediated by immune responses and it takes to destructive cellular mechanism. Matrix metalloproteinases (MMPs) can activate immune response, with extracellular matrix components degradation. These mechanisms could lead to CHD.
Xie 2015 [23]	*P. gingivalis* biology	*P. gingivalis* outer membrane produces vesicles. These vesicles can communicate with other member of microbial biofilm and with host cells.
Wu et al., 2014 [24]	Chronic inflammation and Alzheimer’s disease (AD)	Systemic inflammations are correlated with neuroinflammation and inflammation of the brain. Diabetes [25], cardiovascular disease, or PD could be implicated with AD. Chronic periodontitis can be a significant source of systemic inflammation and so AD too.
Scher et al., 2014 [26]	Periodontitis an immune response	There is a correlation between PD a rheumatoid arthritis (RA). Circulating antibodies against periodontopathic bacteria, as *P. gingivalis*, and their associated inflammatory response have been found in RA affected patients. Smoke habits seems to be a co-associated factor for RA development.
Grover et al., 2014 [27]	Antigenic target for *P. gingivalis*	Microbial genome sequencing and bioinformatics could lead to a vaccine development for *P. gingivalis*.
Brusca et al., 2014 [28]	Microbiome and RA	Presence of bacteria in mucosal surfaces can alter local and systemic immune responses and could cause joint inflammation
Reyes et al., 2013 [29]	Periodontal Bacteria and atherosclerosis	Periodontal bacteria can disseminate from oral cavity to systemic vascular tissue, could live in those tissues and damage cells. These bacteria can induce atherosclerosis.
Michaud 2013 [30]	Bacterial infection and Pancreatic Cancer (PC)	*Helicobacter pylor* may be a risk factor for PC, other bacteria that should be considered is *P. gingivalis*. Periodontal disease and *P. gingivalis* play a role on carcinogenesis of pancreas. Systemic inflammation markers can be found due to oral bacteria in the blood.
Han et al., 2013 [31]	Oral bacteria and extraoral infections	Oral commensals and pathogens could support extra-oral infections and inflammations. Some of these conditions are represented by CVD, adverse pregnancy outcomes, RA, inflammatory bowel disease, colorectal cancer, respiratory infection, and organ inflammation or infections
El-Shinnawi et al., 2013 [32]	Cytokines and coronary heart disease	TNF-alpha gene polymorphism is linked to periodontal attachment loss in patients with CHD. *P. gingivalis* and oral inflammation could be associated to insulin resistance and arthritis too.
Imai et al., 2011 [33]	Periodontal disease and AIDS progression	Epigenetic regulation is involved in maintenance and reactivation of HIV-1 by periodontopathic bacteria.
Huck et al., 2011 [34]	Atherosclerosis risk factor and periodontal disease.	PD negatively influences cardiovascular status. Severe form of Atherosclerosis is associated to *P. gingivalis*.
McNicol et al., 2010 [35]	Bacteriaemias and systemic disease	Atherothrombotic disorders may be due to persistent bacteriaemia and oral infection. Bacteriaemias occur frequently in PD patients. Some bacteria, as *P. gingivalis* can activate platelets in vitro.
Inaba et al., 2010 [36]	Periodontal disease and systemic diseases	Periodontal disease is associated with CVD, preterm delivery of low birth weight, diabetes mellitus, respiratory diseases, and osteoporosis.
Hayashi et al., 2010 [37]	Periodontal disease and systemic diseases	*P. gingivalis* induce a chronic inflammation, this status could lead to infection, that is correlated with systemic diseases as diabetes, preterm birth, stroke, and CVD. *P. gingivalis* stimulate chronic inflammation and plaque accumulation and has a role on Toll-like receptors signaling.
Detert et al., 2010 [38]	Periodontal disease and rheumatoid arthritis	The existence of rheumatic or other inflammatory disease may promote periodontal disease, and periodontal disease maintains systemic diseases. *P. gingivalis* plays an important role on citrullination.

**Table 3:** Medical disease subdivided study.

Authors and Year	Items	Medical Disease
Bale et al., 2017 [18]	Serum lipoprotein concentration, endothelial permeability, lipoprotein binding in intima	Cardiology
Chistiakov et al., 2016 [21]	PD and cardiovascular disease (CVD), age, smoking, diabetes	
Alfakry et al., 2016 [22]	Chronic infection and coronary heart disease (CHD)	
Reyes et al., 2013 [29]	Periodontal bacteria and atherosclerosis	
El-Shinnawi et al., 2013 [32]	Cytokines and coronary heart disease	
Huck et al., 2011 [34]	Atherosclerosis risk factor and periodontal disease.	
McNicol et al., 2010 [35]	Bacteriaemias and systemic disease	
Inaba et al., 2010 [36]	Periodontal disease and systemic diseases	
Hayashi et al., 2010 [37]	Periodontal disease and systemic diseases	
Scher et al., 2014 [26]	Periodontitis an immune response	Rheumatology
Han et al., 2013 [31]	Oral bacteria and extraoral infections	
El-Shinnawi et al., 2013 [32]	Cytokines and coronary heart disease	Diabetology
Gholizadeh et al., 2016 [20]	*P. gingivalis* and oral cancer (OC)	Oncology
Tiantian et al., 2016 [19]	Virus and periodontal disease correlation	Biology and Immunology
Grover et al., 2014 [27]	Antigenic target for *P. gingivalis*	
Michaud 2013 [30]	Bacterial infection and pancreatic cancer (PC)	
Brusca et al., 2014 [28]	Microbiome and RA	
Imai et al., 2011 [33]	Periodontal disease and AIDS progression	
Dominy et al., 2019 [10]	*P. gingivalis* and Alzheimer’s disease	Neurology
Wu et al., 2014 [24]	Chronic inflammation and Alzheimer’s disease (AD)	

**Table 4:** Risk of bias risk.

Authors and Year	Risk of Bias			
	Unclear	Low	Moderate	High
Dominy et al., 2019 [10]			✓	
Bale et al., 2017 [18]			✓	
Tiantian et al., 2016 [19]			✓	
Gholizadeh et al., 2016 [20]		✓		
Chistiakov et al., 2016 [21]			✓	
Alfakry et al., 2016 [22]				✓
Xie 2015 [23]				✓
Wu et al., 2014 [24]			✓	
Scher et al., 2014 [26]				✓
Grover et al., 2014 [27]			✓	
Brusca et al., 2014 [28]			✓	
Reyes et al., 2013 [29]			✓	
Michaud 2013 [30]				✓
Han et al., 2013 [31]		✓		
El-Shinnawi et al., 2013 [32]		✓		
Imai et al., 2011 [33]			✓	
Huck et al., 2011 [34]			✓	
McNicol et al., 2010 [35]			✓	
Inaba et al., 2010 [36]			✓	
Hayashi et al., 2010 [37]			✓	
Detert et al., 2010 [38]			✓	

## References

[1] Putsep K., Carlsson G., Boman H.G., Andersson M. (2002). Deficiency of antibacterial peptides in patients with morbus Kostmann: An observation study. Lancet.

[2] Poole D.F., Newman H.N. (1971). Dental plaque and oral health. Nature.

[3] Pihlstrom B.L., Michalowicz B.S., Johnson N.W. (2005). Periodontal diseases. Lancet.

[4] Holt S.C., Ebersole J., Felton J., Brunsvold M., Kornman K.S. (1988). Implantation of Bacteroides gingivalis in nonhuman primates initiates progression of periodontitis. Science.

[5] Bjertness E., Hansen B.F., Berseth G., Gronnesby J.K. (1993). Oral hygiene and periodontitis in young adults. Lancet.

[6] Hanisch M., Hoffmann T., Bohner L., Hanisch L., Benz K., Kleinheinz J., Jackowski J. (2019). Rare Diseases with Periodontal Manifestations. Int. J. Environ. Res. Public Health.

[7] Mysak J., Podzimek S., Sommerova P., Lyuya-Mi Y., Bartova J., Janatova T., Prochazkova J., Duskova J. (2014). *Porphyromonas gingivalis*: Major periodontopathic pathogen overview. J. Immunol. Res..

[8] Oliveira L., Moraes M.F., Oliveira P., Abecasis P. (1999). A train driver with painful legs. Lancet.

[9] Katz J., Marc H., Porter S., Ruskin J. (2001). Inflammation, periodontitis, and coronary heart disease. Lancet.

[10] Dominy S.S., Lynch C., Ermini F., Benedyk M., Marczyk A., Konradi A., Nguyen M., Haditsch U., Raha D., Griffin C. (2019). *Porphyromonas gingivalis* in Alzheimer’s disease brains: Evidence for disease causation and treatment with small-molecule inhibitors. Sci. Adv..

[11] Olsen I., Yilmaz Ö. (2019). Possible role of *Porphyromonas gingivalis* in orodigestive cancers. J. Oral. Microbiol..

[12] Magana M., Sereti C., Ioannidis A., Mitchell C.A., Ball A.R., Magiorkinis E., Chatzipanagiotou S., Hamblin M.R., Hadjifrangiskou M., Tegos G.P. (2018). Options and Limitations in Clinical Investigation of Bacterial Biofilms. Clin. Microbiol. Rev..

[13] Africa C.W.J., Nel J., Stemmet M. (2014). Anaerobes and bacterial vaginosis in pregnancy: Virulence factors contributing to vaginal colonisation. Int. J. Environ. Res. Public Health.

[14] da Costa Santos C.M., de Mattos Pimenta C.A., Nobre M.R. (2007). The PICO strategy for the research question construction and evidence search. Rev. Lat. Am. Enferm..

[15] Tian J.H., Ge L., Li L. (2015). The PRISMA Extension Statement. Ann. Intern. Med..

[16] Liu H., Zhou X., Yu G., Sun X. (2019). The effects of the PRISMA statement to improve the conduct and reporting of systematic reviews and meta-analyses of nursing interventions for patients with heart failure. Int. J. Nurs. Pr..

[17] Hutton B., Salanti G., Caldwell D.M., Chaimani A., Schmid C.H., Cameron C., Ioannidis J.P., Straus S., Thorlund K., Jansen J.P. (2015). The PRISMA extension statement for reporting of systematic reviews incorporating network meta-analyses of health care interventions: Checklist and explanations. Ann. Intern. Med..

[18] Bale B.F., Doneen A.L., Vigerust D.J. (2017). High-risk periodontal pathogens contribute to the pathogenesis of atherosclerosis. Postgrad. Med. J..

[19] Tiantian M., Xin L. (2016). Promotion of *Porphyromonas gingivalis* to viral disease. Hua Xi Kou Qiang Yi Xue Za Zhi Huaxi Kouqiang Yixue Zazhi West China J. Stomatol..

[20] Gholizadeh P., Eslami H., Yousefi M., Asgharzadeh M., Aghazadeh M., Kafil H.S. (2016). Role of oral microbiome on oral cancers, a review. Biomed. Pharmacother. Biomed. Pharmacother..

[21] Chistiakov D.A., Orekhov A.N., Bobryshev Y.V. (2016). Links between atherosclerotic and periodontal disease. Exp. Mol. Pathol..

[22] Alfakry H., Malle E., Koyani C.N., Pussinen P.J., Sorsa T. (2016). Neutrophil proteolytic activation cascades: A possible mechanistic link between chronic periodontitis and coronary heart disease. Innate Immun..

[23] Xie H. (2015). Biogenesis and function of *Porphyromonas gingivalis* outer membrane vesicles. Future Microbiol..

[24] Wu Z., Nakanishi H. (2014). Connection between periodontitis and Alzheimer’s disease: Possible roles of microglia and leptomeningeal cells. J. Pharmacol. Sci..

[25] Cervino G., Terranova A., Briguglio F., De Stefano R., Famà F., D’Amico C., Amoroso G., Marino S., Gorassini F., Mastroieni R. (2019). Diabetes: Oral health related quality of life and oral alterations. BioMed Res. Int..

[26] Scher J.U., Bretz W.A., Abramson S.B. (2014). Periodontal disease and subgingival microbiota as contributors for rheumatoid arthritis pathogenesis: Modifiable risk factors?. Curr. Opin. Rheumatol..

[27] Grover V., Kapoor A., Malhotra R., Kaur G. (2014). *Porphyromonas gingivalis* antigenic determinants-potential targets for the vaccine development against periodontitis. Infect. Disord. Drug Targets.

[28] Brusca S.B., Abramson S.B., Scher J.U. (2014). Microbiome and mucosal inflammation as extra-articular triggers for rheumatoid arthritis and autoimmunity. Curr. Opin. Rheumatol..

[29] Reyes L., Herrera D., Kozarov E., Roldan S., Progulske-Fox A. (2013). Periodontal bacterial invasion and infection: Contribution to atherosclerotic pathology. J. Clin. Periodontol..

[30] Michaud D.S. (2013). Role of bacterial infections in pancreatic cancer. Carcinogenesis.

[31] Han Y.W., Wang X. (2013). Mobile microbiome: Oral bacteria in extra-oral infections and inflammation. J. Dent. Res..

[32] El-Shinnawi U., Soory M. (2013). Associations between periodontitis and systemic inflammatory diseases: Response to treatment. Recent Pat. Endocr. Metab. Immune Drug Discov..

[33] Imai K., Ochiai K. (2011). Role of histone modification on transcriptional regulation and HIV-1 gene expression: Possible mechanisms of periodontal diseases in AIDS progression. J. Oral Sci..

[34] Huck O., Saadi-Thiers K., Tenenbaum H., Davideau J.L., Romagna C., Laurent Y., Cottin Y., Roul J.G. (2011). Evaluating periodontal risk for patients at risk of or suffering from atherosclerosis: Recent biological hypotheses and therapeutic consequences. Arch. Cardiovasc. Dis..

[35] McNicol A., Israels S.J. (2010). Mechanisms of oral bacteria-induced platelet activation. Can. J. Physiol. Pharmacol..

[36] Inaba H., Amano A. (2010). Roles of oral bacteria in cardiovascular diseases—From molecular mechanisms to clinical cases: Implication of periodontal diseases in development of systemic diseases. J. Pharmacol. Sci..

[37] Hayashi C., Gudino C.V., Gibson F.C., Genco C.A. (2010). Review: Pathogen-induced inflammation at sites distant from oral infection: Bacterial persistence and induction of cell-specific innate immune inflammatory pathways. Mol. Oral Microbiol..

[38] Detert J., Pischon N., Burmester G.R., Buttgereit F. (2010). The association between rheumatoid arthritis and periodontal disease. Arthritis Res. Ther..

[39] Fiorillo L. (2019). Oral Health: The First Step to Well-Being. Medicina.

[40] Lindhe J. (2009). Parodontologia Clinica E Implantologia Orale.

[41] Lombardi T., Bernardello F., Berton F., Porrelli D., Rapani A., Camurri Piloni A., Fiorillo L., Di Lenarda R., Stacchi C. (2018). Efficacy of Alveolar Ridge Preservation after Maxillary Molar Extraction in Reducing Crestal Bone Resorption and Sinus Pneumatization: A Multicenter Prospective Case-Control Study. BioMed Res. Int..

[42] Isola G., Cicciù M., Fiorillo L., Matarese G. (2017). Association between odontoma and impacted teeth. J. Craniofac. Surg..

[43] Cervino G., Romeo U., Lauritano F., Bramanti E., Fiorillo L., D’Amico C., Milone D., Laino L., Campolongo F., Rapisarda S. (2018). Fem and Von Mises Analysis of OSSTEM(r) Dental Implant Structural Components: Evaluation of Different Direction Dynamic Loads. Open Dent. J..

[44] Bramanti E., Matacena G., Cecchetti F., Arcuri C., Cicciù M. (2013). Oral health-related quality of life in partially edentulous patients before and after implant therapy: A 2-year longitudinal study. ORAL Implantol..

[45] Lo Giudice G., Cutroneo G., Centofanti A., Artemisia A., Bramanti E., Militi A., Rizzo G., Favaloro A., Irrera A., Lo Giudice R. (2015). Dentin morphology of root canal surface: A quantitative evaluation based on a scanning electronic microscopy study. BioMed Res. Int..

[46] Germano F., Bramanti E., Arcuri C., Cecchetti F., Cicciù M. (2013). Atomic force microscopy of bacteria from periodontal subgingival biofilm: Preliminary study results. Eur. J. Dent..

[47] Maiorana C., Beretta M., Grossi G.B., Santoro F., Herford A.S., Nagursky H., Cicciù M. (2011). Histomorphometric evaluation of anorganic bovine bone coverage to reduce autogenous grafts resorption: Preliminary results. Open Dent. J..

[48] Luongo G., Oteri G. (2010). A noninterventional study documenting use and success of implants with a new chemically modified titanium surface in daily dental practice. J. Oral Implantol..

[49] Herford A.S., Lu M., Akin L., Cicciù M. (2012). Evaluation of a porcine matrix with and without platelet-derived growth factor for bone graft coverage in pigs. Int. J. Oral Maxillofac. Implant..

[50] Cervino G., Fiorillo L., Herford A.S., Romeo U., Bianchi A., Crimi S., D’Amico C., De Stefano R., Troiano G., Santoro R. (2019). Molecular Biomarkers Related to Oral Carcinoma: Clinical Trial Outcome Evaluation in a Literature Review. Dis. Markers.

[51] Crimi S., Fiorillo L., Bianchi A., D’Amico C., Amoroso G., Gorassini F., Mastroieni R., Marino S., Scoglio C., Catalano F. (2019). Herpes Virus, Oral Clinical Signs and QoL: Systematic Review of Recent Data. Viruses.

[52] Cervino G., Fiorillo L., Monte I.P., De Stefano R., Laino L., Crimi S., Bianchi A., Herford A.S., Biondi A., Cicciù M. (2019). Advances in Antiplatelet Therapy for Dentofacial Surgery Patients: Focus on Past and Present Strategies. Materials.

[53] Öğrendik M. (2017). Periodontal Pathogens in the Etiology of Pancreatic Cancer. Gastrointest. Tumors.

